# 223. Development and Analysis of a Novel DOOR Endpoint for Complicated Intra-abdominal Infections (cIAI) Using 10 Registrational Trials for Antibacterial Drugs

**DOI:** 10.1093/ofid/ofac492.301

**Published:** 2022-12-15

**Authors:** Tori Kinamon, Ramya Gopinath, Ursula Waack, Mark Needles, Daniel Rubin, Deborah Collyar, Sarah B Doernberg, Scott R Evans, Toshimitsu Hamasaki, Thomas L Holland, Jessica Howard-Anderson, Henry Chambers, Vance G Fowler, Sumathi Nambiar, Peter Kim, Helen W Boucher

**Affiliations:** FDA, Durham, North Carolina; Food and Drug Administration, Silver Spring, Maryland; Food and Drug Administration, Silver Spring, Maryland; FDA, Durham, North Carolina; FDA, Durham, North Carolina; Patient Advocates In Research (PAIR), Danville, California; University of California, San Francisco, San Francisco, California; Milken Institute School of Public Health, Rockville, Maryland; George Washington University, Rockville, Maryland; Duke University Medical Center, Durham, North Carolina; Emory University, Atlanta, Georgia; University of California San Francisco, San Francisco, California; Duke University Medical Center, Durham, North Carolina; Johnson and Johnson, Germantown, Maryland; US FDA, Silver Spring, Maryland; Tufts Medical Center, Boston, Massachusetts

## Abstract

**Background:**

Desirability of outcome ranking (DOOR) uses an ordinal ranking system to evaluate global outcomes in clinical trial participants by incorporating safety and efficacy assessments into a single endpoint. In this study, we developed and applied a DOOR endpoint for cIAI clinical trials.

**Methods:**

We reviewed 10 Phase 3 noninferiority trials for cIAI with electronic patient-level data (n=5473 participants) submitted to the FDA between 2005-2021. Extending previous work [CID. 2019:68(10):1691-8)], we developed an expanded cIAI-specific DOOR endpoint based on clinically meaningful events captured in trial datasets and those that were unique to patients with cIAI. Using this DOOR endpoint, we assigned each participant a DOOR rank, estimated the probability that a participant in the study treatment arm in each trial would have a more desirable DOOR rank than if assigned to the comparator arm, and analyzed individual components of clinical experience in each trial.

**Results:**

Based on analysis of available data, we noted heterogeneity in definitions of “indeterminate” clinical outcomes, and significant diversity and increased incidence of infectious complications (ICs), serious adverse events (SAEs), and surgical/percutaneous procedures in participants without clinical cure. These informed the expansion of the DOOR endpoint for cIAI to include clinical efficacy outcomes, ICs, SAEs, and additional procedures (Table 1). The DOOR distributions between treatment and comparator arms in all 10 trials were similar. DOOR probability estimates for the 10 trials ranged from 44.5% to 50.3% but were not nominally statistically significant. Component analyses in two trials showed that the study treatment was nominally statistically inferior to the comparator with regard to SAEs and clinical failure, respectively (Fig. 1b, 1c).

**Table 1.**
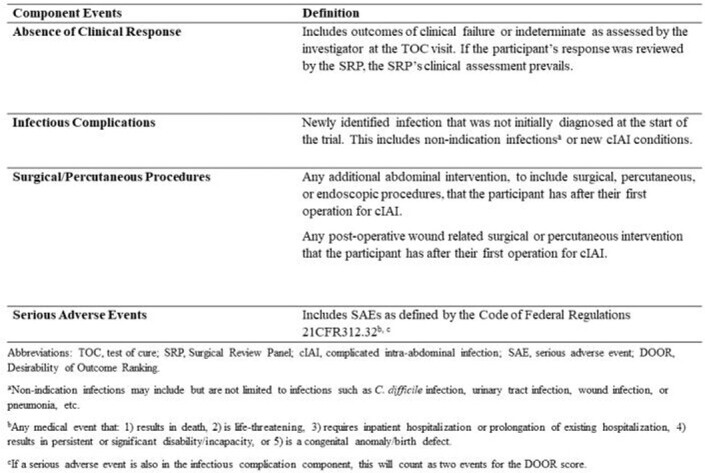
cIAI-Specific DOOR Endpoint

**Figure 1.**
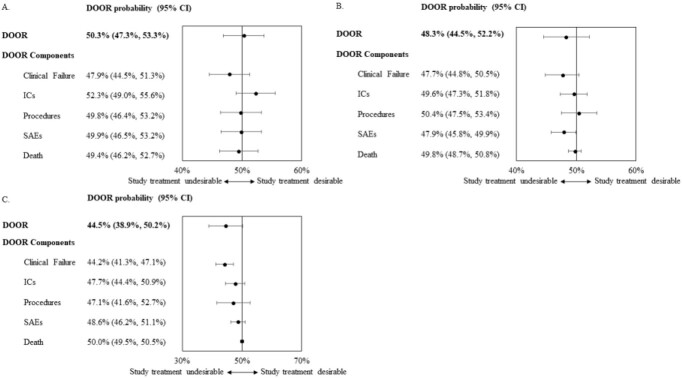
Forest plot listing the DOOR probabilities and probability for each DOOR component from 3 trials.

Trial 1 has no significant differences between the treatment arms in the component analysis (A). The study treatment arm was shown to be nominally statistically inferior for SAEs in Trial 2 (B) and for clinical failure in Trial 3 (C).

**Conclusion:**

We developed a cIAI-specific DOOR endpoint to better elucidate the events that participants experienced in these trials. The component analysis allowed more nuanced evaluation of the factors that contributed to the composite DOOR probability estimate and provided a visual display of the risk-benefit assessment of a study treatment vs. the comparator. Our study was limited by its retrospective approach and trial design heterogeneity.

**Disclosures:**

**Deborah Collyar, B.Sci**, Apellis Pharmaceuticals, Inc.: Advisor/Consultant|Kinnate Biopharma: Advisor/Consultant|M2GEN: Advisor/Consultant|Maxis Clinical Sciences: Advisor/Consultant|Parexel: Honoraria|Pfizer: Honoraria|Roundtable Analytics, Inc.: Ownership Interest **Sarah B. Doernberg, MD, MAS**, Basilea: Clincal events committee|Genentech: Advisor/Consultant|Gilead: Grant/Research Support|Regeneron: Grant/Research Support|Shinogi: Clincal events committee **Scott R. Evans, Ph.D., M.S.**, Abbvie: DSMB|Akouos: DSMB|Apellis: DSMB|AstraZeneca: Advisor/Consultant|Atricure: Advisor/Consultant|Becton Dickenson: Advisor/Consultant|Breast International Group: DSMB|Candel: DSMB|ChemoCentrix: Advisor/Consultant|Clover: DSMB|DayOneBio: DSMB|DeGruyter: Editor|Duke University: DSMB|Endologix: Advisor/Consultant|FHI Clinical: DSMB|Genentech: Advisor/Consultant|Horizon: Advisor/Consultant|International Drug Development Institute: Advisor/Consultant|Janssen: Advisor/Consultant|Lung Biotech: DSMB|Neovasc: Advisor/Consultant|NIH: Grant/Research Support|Nobel Pharma: Advisor/Consultant|Nuvelution: DSMB|Pfizer: DSMB|Rakuten: DSMB|Roche: DSMB|Roivant: Advisor/Consultant|SAB Biopharm: DSMB|SVB Leerink: Advisor/Consultant|Takeda: DSMB|Taylor & Francis: Book royalties|Teva: DSMB|Tracon: DSMB|University of Penn: DSMB|Vir: DSMB **Thomas L. Holland, MD**, Aridis: Advisor/Consultant|Lysovant: Advisor/Consultant **Henry Chambers, MD**, Merck: DSMB member|Merck: Stocks/Bonds|Moderna: Stocks/Bonds **Vance G. Fowler, Jr, MD, MHS**, Affinergy: Grant/Research Support|Affinergy: Honoraria|Affinium: Honoraria|Amphliphi Biosciences: Honoraria|ArcBio: Stocks/Bonds|Basilea: Grant/Research Support|Basilea: Honoraria|Bayer: Honoraria|C3J: Honoraria|Cerexa/Forest/Actavis/Allergan: Grant/Research Support|Contrafect: Grant/Research Support|Contrafect: Honoraria|Cubist/Merck: Grant/Research Support|Debiopharm: Grant/Research Support|Deep Blue: Grant/Research Support|Destiny: Honoraria|Genentech: Grant/Research Support|Genentech: Honoraria|Integrated Biotherapeutics: Honoraria|Janssen: Grant/Research Support|Janssen: Honoraria|Karius: Grant/Research Support|Medicines Co.: Honoraria|MedImmune: Grant/Research Support|MedImmune: Honoraria|NIH: Grant/Research Support|Novartis: Grant/Research Support|Novartis: Honoraria|Pfizer: Grant/Research Support|Regeneron: Grant/Research Support|Regeneron: Honoraria|Sepsis diagnostics: Sepsis diagnostics patent pending|UpToDate: Royalties|Valanbio: Stocks/Bonds **Sumathi Nambiar, MD MPH**, Johnson and Johnson: Stocks/Bonds **Helen W. Boucher, MD**, American Society of Microbiology: Honoraria|Elsevier: Honoraria|Sanford Guide: Honoraria.

